# Changes in the Etiology of Acute Respiratory Infections among Children in Novosibirsk, Russia, between 2019 and 2022: The Impact of the SARS-CoV-2 Virus

**DOI:** 10.3390/v15040934

**Published:** 2023-04-09

**Authors:** Olga G. Kurskaya, Elena A. Prokopyeva, Ivan A. Sobolev, Mariya V. Solomatina, Tereza A. Saroyan, Nikita A. Dubovitskiy, Anastasiya A. Derko, Alina R. Nokhova, Angelika V. Anoshina, Natalya V. Leonova, Olga A. Simkina, Tatyana V. Komissarova, Alexander M. Shestopalov, Kirill A. Sharshov

**Affiliations:** 1 Laboratory of Molecular Epidemiology and Biodiversity of Viruses, Federal Research Center of Fundamental and Translational Medicine, Novosibirsk 630060, Russia; 2 Department of Children’s Diseases, Novosibirsk Children’s Municipal Clinical Hospital No 6, Novosibirsk 630015, Russia; 3 Department of Children’s Diseases, Novosibirsk Children’s Municipal Clinical Hospital No 3, Novosibirsk 630040, Russia

**Keywords:** HIFV, HRSV, HCoV, HPIV, HMPV, HRV, HAdV, HBoV, SARS-CoV-2

## Abstract

A wide range of human respiratory viruses are known that may cause acute respiratory infections (ARIs), such as influenza A and B viruses (HIFV), respiratory syncytial virus (HRSV), coronavirus (HCoV), parainfluenza virus (HPIV), metapneumovirus (HMPV), rhinovirus (HRV), adenovirus (HAdV), bocavirus (HBoV), and others. The severe acute respiratory syndrome coronavirus 2 (SARS-CoV-2) caused the COronaVIrus Disease (COVID) that lead to pandemic in 2019 and significantly impacted on the circulation of ARIs. The aim of this study was to analyze the changes in the epidemic patterns of common respiratory viruses among children and adolescents hospitalized with ARIs in hospitals in Novosibirsk, Russia, from November 2019 to April 2022. During 2019 and 2022, nasal and throat swabs were taken from a total of 3190 hospitalized patients 0–17 years old for testing for HIFV, HRSV, HCoV, HPIV, HMPV, HRV, HAdV, HBoV, and severe acute respiratory syndrome coronavirus 2 (SARS-CoV-2) by real-time PCR. The SARS-CoV-2 virus dramatically influenced the etiology of acute respiratory infections among children and adolescents between 2019 and 2022. We observed dramatic changes in the prevalence of major respiratory viruses over three epidemic research seasons: HIFV, HRSV, and HPIV mainly circulated in 2019–2020; HMPV, HRV, and HCoV dominated in 2020–2021; and HRSV, SARS-CoV-2, HIFV, and HRV were the most numerous agents in 2021–2022. Interesting to note was the absence of HIFV and a significant reduction in HRSV during the 2020–2021 period, while HMPV was absent and there was a significant reduction of HCoV during the following epidemic period in 2021–2022. Viral co-infection was significantly more frequently detected in the 2020–2021 period compared with the other two epidemic seasons. Certain respiratory viruses, HCoV, HPIV, HBoV, HRV, and HAdV, were registered most often in co-infections. This cohort study has revealed that during the pre-pandemic and pandemic periods, there were dramatic fluctuations in common respiratory viruses registered among hospitalized patients 0–17 years old. The most dominant virus in each research period differed: HIFV in 2019–2020, HMPV in 2020–2021, and HRSV in 2021–2022. Virus–virus interaction was found to be possible between SARS-CoV-2 and HRV, HRSV, HAdV, HMPV, and HPIV. An increase in the incidence of COVID-19 was noted only during the third epidemic season (January to March 2022).

## 1. Introduction

Respiratory viruses are a leading cause of acute respiratory infection (ARI) in both children and adolescents [1,2]. A wide range of respiratory viruses cause ARIs, such as human influenza A and B viruses (HIFV), human respiratory syncytial virus (HRSV), human alphacoronaviruses (NL63/229E), betacoronaviruses (OC43/HKU1) (HCoV), human parainfluenza virus types 1–4 (HPIV), human metapneumoviruses (HMPV), human rhinovirus (HRV), human adenovirus (HAdV), human bocavirus (HBoV), and others. The detection of respiratory tract diseases caused by more than one virus occurs in 35% of cases [3,4]. Since the surge of severe acute respiratory syndrome coronavirus 2 (SARS-CoV-2) that caused the COVID-19 pandemic, several studies have shown that the epidemiological and clinical features of ARI infections changed [5,6]. However, the clinical manifestation of viral co-infections and the significance of such respiratory diseases have not yet been fully studied.

In the present study, we analyzed data of disease severity and specimens from children aged 0–17 hospitalized with ARIs in Novosibirsk, Russia, from November 2019 to April 2022. We collected statistical data from 3190 patients from whom nasal and throat swabs were tested for HIFV, HRSV, HCoV, HPIV, HMPV, HRV, HAdV, HBoV, and SARS-CoV-2 to assess the virus prevalence, age distribution, variability of co-infections, and other epidemiological characteristics of common ARIs that affect children. The data obtained may be important for the prediction of disease progression, prognosis, and treatment strategies, especially among high-risk groups such as young children.

## 2. Materials and Methods

### 2.1. Ethics Issues

All aspects of the study were approved by the Committee on Biomedical Ethics of the Federal Research Center of Fundamental and Translational Medicine Protocol No. 3 of 28 January 2019. Written informed consent was obtained from all parents/legal guardians prior to sample taking.

### 2.2. Sample Collection

Nasal and throat swabs were taken from children (aged 0–17 years) hospitalized with symptoms of acute respiratory infections during three successive research periods: the first being 2019–2020 (November–April), which was the pre-pandemic season; the second was 2020–2021 (November–April), and the third was 2021–2022 (October–April). Both of the latter were seasons of the continuing pandemic. We enrolled children who had at least one of the systemic symptoms (fever, headache, myalgia, or malaise) and one of the respiratory symptoms (cough, rhinorrhea, nasal congestion, sore throat, shortness of breath, lung auscultation abnormalities, chest pain). Samples were taken in two hospitals, Novosibirsk Children’s Municipal Clinical Hospital No. 6 and Novosibirsk Children’s Municipal Clinical Hospital No. 3. Samples obtained from the nose or throat were immediately prepared for analysis. If they were not analyzed immediately, samples were stored at 2 °C to 8 °C for up to four hours.

### 2.3. Virus Detection

Nasal and throat swabs were taken from all patients within 24 h of admission and tested using real-time polymerase chain reaction (RT-PCR) (AmpliSens ARVI-screen-FL and AmpliPrime Influenza SARS-CoV-2/Flu(A/B/H1pdm09) RT-PCR Kits (Interlabservice, Moscow, Russia) for SARS-CoV-2 and other respiratory viruses such as influenza A and B viruses (HIFV); respiratory syncytial virus (HRSV); alphacoronaviruses (NL63/229E) and betacoronaviruses (OC43/HKU1) (HCoV); parainfluenza virus types 1–4 (HPIV); metapneumovirus (HMPV); rhinovirus (HRV); adenovirus (HAdV); and bocavirus (HBoV). Positive and negative controls were included in each run.

### 2.4. Statistical Analysis of the Viral Data and Clinical Features of ARI

Data analysis was performed using Microsoft Excel 16.16.2 for MacOs and GraphPad 9.1.1. The baseline characteristics of all positive viral detections were analyzed using a two-tailed Chi-square test (two by two table) undertaken to compare infection rates for respiratory viruses among different age groups; a *p*-value < 0.05 was considered to be statistically significant.

In addition, each hospital provided information on their patients through their diagnostic assays. Data were collected on sex, age, selected common risk factors for respiratory infections (e.g., the presence of signs of upper respiratory tract infections—often in multiple locations); pneumonia; bronchitis; bronchiolitis; hyperthermia; hypoxia; admission to an intensive care unit (ICU)), treatment; and days of hospitalization. The subjects were divided into three age groups: 0–2 years, 3–6 years, 7–17 years, as well as an aggregated 0–17-year age group.

## 3. Results

### 3.1. Age and Gender Distribution of Patients with Acute Respiratory Infections before and during the COVID-19 Pandemic

**Figure 1:**
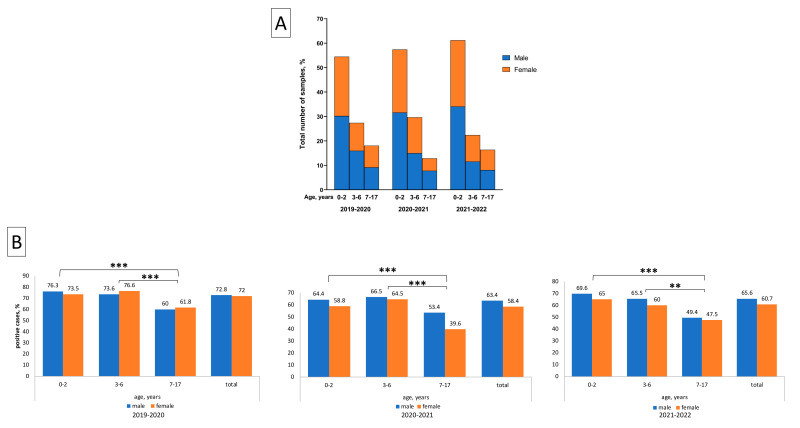
Age and gender distribution data from a total of 3190 children and adolescents hospitalized with acute respiratory infections in 2019–2022. (**A**) PCR-confirmed samples from children aged 0–17 years (**B**) Age and gender distribution data by the three successive research periods: the first being 2019–2020), which was the pre-pandemic season; the second was 2020–2021, and the third was 2021–2022. *** and **: Differences between groups were statistically significant (Chi-square test, *p* < 0.01 and *p* < 0.05, respectively).

A total of 3190 children aged 0–17 years were included in the study over the 2019–2022 period: 1088 patients in 2019–2020, 1130 patients in 2020–2021, and 972 patients in 2021–2022. About 55% (1742/3190) of samples were obtained from boys and 45% (1448/3190) from girls. In this research cohort, no significant gender differences among patients were observed. The majority of samples—57.6% (1836/3190)—were obtained from children aged 0–2 years compared to the other age groups (Chi-square test, *p* < 0.01). The age and sex distribution of patients did not significantly differ within the three epidemic research seasons (Figure 1A).

### 3.2. Detection Rate of Respiratory Viruses

The detection rates of common respiratory viruses for all samples collected in this research were comparatively assessed. The result obtained in 2019–2020 was significantly higher (788/1088 (72.4%)) compared to that for 2020–2021 (691/1130 (61.2%), χ^2^ = 31.72, *p* < 0.05) and for 2021–2022 (616/972 (63.4%), χ^2^ = 19.38, *p* < 0.05) (Figure 1B). The level of virus detection was significantly lower in the 7–17-year age group compared to the 0–2- and 3–6-year age groups (Figure 1B). No gender differences were detected.

The influenza virus was the dominant infectious agent among all viruses studied during the 2019–2020 period (28.7%), followed by respiratory syncytial virus (21.1%). Other viral infections represented were HPIV (9.2%), HRV (7.6%), HBoV (5.6%), HAdV (3.9%), HCoV (2.9%), and HMPV (1.6%). No cases of SARS-CoV-2 infection among children or adolescents were detected during the 2019–2020 period.

During the following research period (2021–2022), the etiology of ARIs changed dramatically. From November 2020 to April 2021, no cases of influenza were detected in the patients examined. Respiratory syncytial virus was found in only three patients (i.e., 0.2%). Metapneumovirus turned out to be the leading etiological agent of ARI, accounting for 28% of the tested samples. The next most common viruses were rhinovirus, detected in 16% of cases, and human coronaviruses (NL63, 229E, HKU1, OC43), detected in 13% of cases. Parainfluenza viruses were detected in 9.7% of cases, while bocavirus was detected in 3.9%, and adenovirus in 3.5% of cases. The new coronavirus—SARS-CoV-2—was identified in only 0.2% of the children examined.

**Figure 2:**
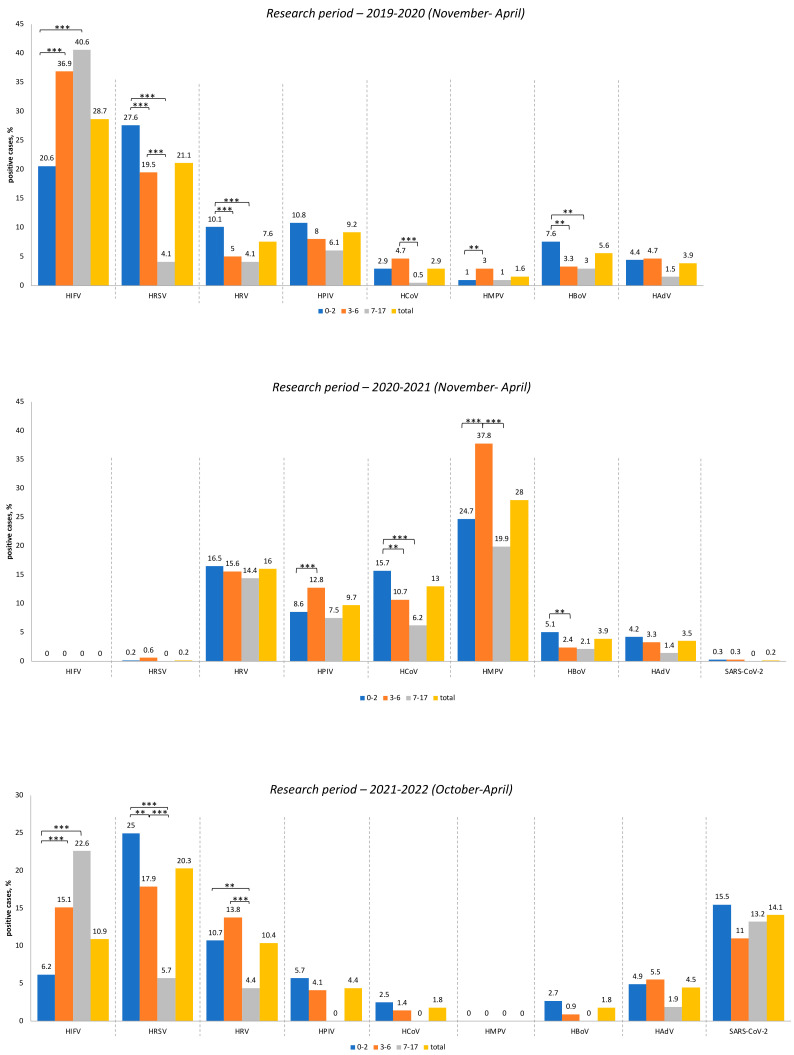
Viral load among various child age groups between 2019 and 2022. All data are presented as a percentage of the positive cases relative to the total number of PCR-confirmed samples received from patients during the research period from 2019–2022. Abbreviations: HIFV—influenza A and B viruses; HRSV—respiratory syncytial virus; HRV—rhinovirus; HPIV—parainfluenza virus types 1–4; HCoV—alphacoronaviruses (NL63/229E) and betacoronaviruses (OC43/HKU1); HMPV—metapneumovirus; HBoV—bocavirus; HAdV—adenovirus; and SARS-CoV-2—severe acute respiratory syndrome coronavirus 2. *** and **—Differences between groups were statistically significant (Chi-square test, *p* < 0.01 and *p* < 0.05, respectively).

During the third research period (2021–2022), the influenza virus re-emerged and was detected in 10.9% of patients. We detected only the subtype H3N2 of the influenza A virus. Interestingly, the etiological leader among the ARI was the human respiratory syncytial virus, which was found in 20.3% of cases examined during this period. By this period, the number of patients infected with SARS-CoV-2 had increased to 14.1%. Rhinovirus was detected in 10.4% of children. Other respiratory viruses were detected in less than 5% of cases: HAdV (4.5%), HPIV (4.4%), HCoV (1.8%), and HBoV (1.8%). It should be noted that we did not detect any case of human metapneumovirus during the whole 2021–2022 epidemic period. The viral load among various child age groups between 2019 and 2022 and a comparative analysis are shown in Figure 2 and Supplementary Table S1, respectively.

### 3.3. The Level of Virus Detection in Different Age Groups

#### 3.3.1. Research Period—2019–2020

By comparing the level of virus detection in different age groups, it was shown that the proportion of influenza virus significantly increased the older the child age group: (*p* < 0.01, χ^2^ = 30.29 for 0–2 years vs. 3–6 years; *p* < 0.01, χ^2^ =31.19 for 0–2 years vs. 7–17 years) (Figure 2). The level of detection of respiratory syncytial virus significantly decreased the older the child age group (*p* < 0.01, χ^2^ =7.12 for 0–2 years vs. 3–6 years; *p* < 0.01, χ^2^ = 201.5 for 3–6 years vs. 7–17 years; *p* < 0.01, χ^2^ =48.34 for 0–2 years vs. 7–17 years). It was noted that seasonal coronaviruses (NL63, 229E, OC43, HKU1) and metapneumovirus reached a maximum in the 3–6-year age group (Chi-square test, *p* < 0.01 and *p* < 0.05), while rhinovirus and bocavirus were most often detected in patients in the 0–2-year age group (Chi-square test, *p* < 0.01 and *p* < 0.05). No age differences were detected when children were infected with agents such as parainfluenza virus types 1–4 and adenovirus.

#### 3.3.2. Research Period—2020–2021

By comparing common respiratory virus incidences among infected children belonging to different age groups it was shown that the proportion of metapneumovirus was significantly higher among patients aged 3–6 years (*p* < 0.01, χ^2^ = 13.48 for 0–2 years vs. 3–6 years; *p* < 0.01, χ^2^ = 14.95 for 3–6 years vs. 7–17 years) (Figure 2). The detection level of seasonal coronaviruses (NL63, 229E, OC43, HKU1) significantly decreased the older the child age group (*p* < 0.05, χ^2^ = 4.64 for 0–2 years vs. 3–6 years; *p* < 0.01, χ^2^ = 9.09 for 0–2 years vs. 7–17 years). As to the effects of parainfluenza virus types 1–4, the maximum viral load was recorded among children aged 3–6 years (*p* < 0.01, χ^2^ = 4.22 for 0–2 years vs. 3–6 years). The bocavirus was most often detected among patients aged 0–2 years (*p* < 0.05, χ^2^ = 4.07 for 0–2 years vs. 3–6 years). We did not find any differences in cases of infection with adenovirus or SARS-CoV-2 among children of various age groups.

#### 3.3.3. Research Period—2021–2022

The proportion of the influenza virus significantly increased the older the child age group (*p* < 0.01, χ^2^ = 16.13 for 0–2 years vs. 3–6 years; *p* < 0.01, χ^2^ = 38.7 for 0–2 years vs. 7–17 years (Figure 2): a similar result had been registered earlier in the initial 2019–2020 research period. The detection levels of respiratory syncytial virus and rhinovirus significantly decreased with age (Chi-square test, *p* < 0.05 and *p* < 0.01); the same situation was observed during the first research period (2019–2020). No differences among age groups due to the influence of adenovirus or SARS-CoV-2 were detected. It is noteworthy that parainfluenza virus, seasonal coronaviruses, and bocavirus were not registered among patients older than 7 years.

### 3.4. Seasonal Distribution of the Most Common Acute Respiratory Viruses

We analyzed the spread of common respiratory viruses during the three research periods from 2019 to 2022.

In 2019–2020, respiratory syncytial virus was detected from November 2019 to April 2020 with an activity peak in December 2019 (29.3%) and January 2020 (33.1%). The influenza virus was identified from January to April 2020 with the maximum detection levels in February (51.0%) and March 2020 (49.0%).

During the subsequent research period (2020–2021), in the absence of respiratory syncytial virus and influenza virus, we observed a significantly higher detection rate of human metapneumovirus compared to the other two periods: overall, it was detected in 28% of cases, with a peak of activity in March 2021 with a detection rate of 53.3%.

Interestingly, in the last research period (2021–2022), we again found respiratory syncytial virus starting in October 2021 with a detection rate of 63.2%, gradually decreasing to 8–9% in February–April 2022. The influenza virus re-emerged in the 2021–2022 period. Moreover, the onset of influenza incidence was earlier than usual with the first cases detected in October 2021. A peak of activity was observed in November–December 2021: since the third week of January 2022, we did not find any cases of influenza. It is noteworthy, that during this period (2021–2022), we did not find any case of metapneumovirus.

Rhinovirus was detected during all three research seasons, and we observed a slight increase in activity in the 2020–2021 season.

**Figure 3:**
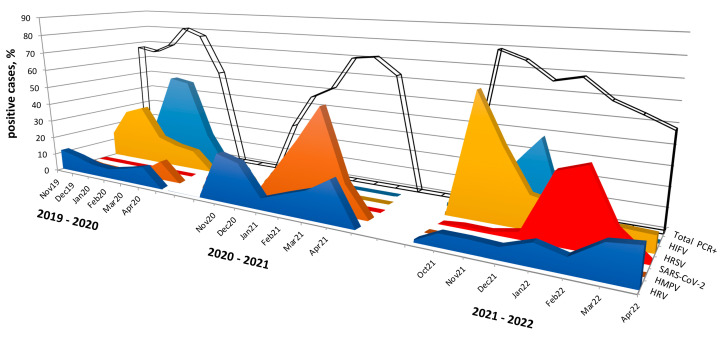
Most common acute respiratory virus fluctuations during 2019–2022. “Total PCR+” refers to the data that are presented as a percentage of the positive cases of PCR-confirmed samples from patients during the three research periods (2019–2022); HIFV—influenza A and B viruses; HRSV—respiratory syncytial virus; SARS-CoV-2—severe acute respiratory syndrome coronavirus 2, HMPV—metapneumovirus; and HRV—rhinovirus.

Despite the widespread incidence of SARS-CoV-2, we did not identify any cases in March–April 2020. During the 2020–2021 season, we detected SARS-CoV-2 in only 0.2% of cases, while in the 2021–2022 season, the SARS-CoV-2 detection rate averaged 14.1%, with maximum activity observed in January and February 2022, amounting to 33.8% and 38.4%, respectively. Such a low level of virus detection in the first two research periods (2019–2021) may be associated with the low incidence of COVID-19 among the child population at the beginning of the pandemic. A significant increase in incidence was observed in January and February 2022 and was associated with the spread of the Omicron variant. The seasonal spread of respiratory viruses among children in Novosibirsk is shown in Figure 3.

### 3.5. Viral Co-Infections

**Figure 4:**
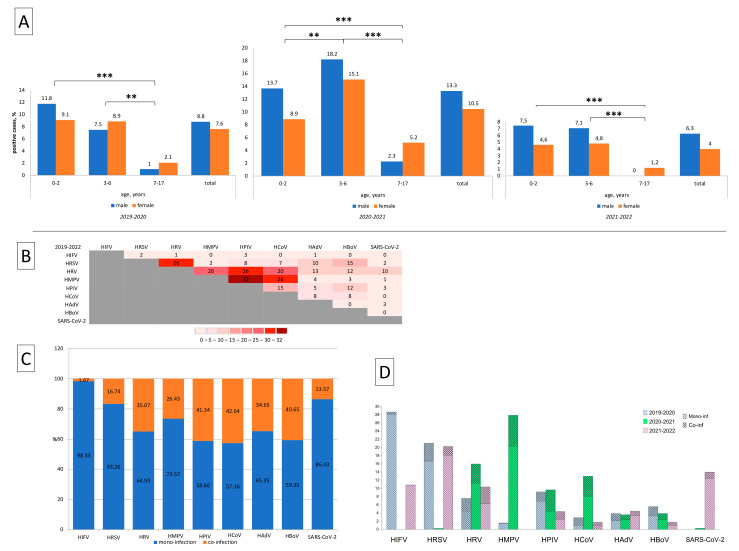
Incidence of co-infections among hospitalized patients aged 0–17 years during 2019–2022. (**A**) Age and gender distribution data of co-infected children and adolescents. (**B**) This figure displays the observed co-infection for each pairing. The co-infection data are plotted as a heat map to aid the visualization of any seasonal trends for each virus during 2019–2022, across all sites. All data are presented as the numerical data of the analyzed medical records of patients who were positive for co-infections during the three research periods. Abbreviations for (**B**–**D**): HIFV—influenza A and B viruses; HRSV—respiratory syncytial virus; HRV—rhinovirus; HPIV—parainfluenza virus types 1–4; HCoV—alphacoronaviruses (NL63/229E) and betacoronaviruses (OC43/HKU1); HMPV—metapneumovirus; HBoV—bocavirus; HAdV—adenovirus; and SARS-CoV-2—severe acute respiratory syndrome coronavirus 2. (**D**) Mono-inf—mono-infection; Co-inf—co-infections. Numbers represent relative frequencies (%). *** and **—Differences between groups were statistically significant (Chi-square test, *p* < 0.01 and *p* < 0.05, accordingly).

Viral co-infections were significantly more frequently detected during the second research period (2020–2021), amounting to 12% (136/1130) of the patients examined, while during the first research period (2019–2020), they were found in 8.3% (90/1088) of patients (χ2 = 8.58, *p* < 0.05), and during the third research period (2021–2022), they amounted to 5.2% (51/972) of patients (χ2 = 29.71, *p* < 0.05). The incidence of co-infections decreased the older the child age group, and the data showed that it was lowest among patients aged 7–17 years in every season (Chi-square test, *p* < 0.05). No significant gender differences in co-infection frequency were found (Figure 4A).

Total: during 2019–2022, the most frequently dual viral combinations were represented by HMPV + HPIV (32 cases), HRSV + HRV, HPIV + HRV, and HMPV + HCoV (26 cases in each combination) (Figure 4B).

It is interesting to note the incidence of triple viral combinations. In 2019–2020, several multi co-infections were registered, such as (HRSV + HRV + HCoV), (HRV + HBoV + HCoV), (HPIV + HBoV + HCoV), (HPIV + HAdV + HCoV), and (HPIV + HBoV + HRV). All of the above triple viral combinations were detected once only. During the following research period (2020–2021), completely different multi co-infections were registered. Some of them were repeated many times, for example (HMPV + HPIV + HCoV) seven times; and (HMPV + HRV + HCoV), (HMPV + HCoV + HBoV), (HMPV + HPIV + HRV) and (HRV + HPIV + HBoV) twice; while only once were the triple viral combinations of (HRV + HCoV + HBoV) and (HRV + HPIV + HCoV) registered. Thus, during the COVID-19 pandemic period, an increase in co-infections in different multi variant (double and triple) viral combinations was recorded. It is noteworthy that we did not detect any triple co-infections with SARS-CoV-2. During the last research period (2021–2022) a few multi viral combinations were found: (HRSV + HCoV + HAdV), (SARS-CoV-2 + HRSV + HAdV), and (SARS-CoV-2 + HRSV + HRV). The last three multi viral co-infections were recorded only once.

The comparative analysis of the frequency of the formation of virus–virus interactions revealed that HRSV and HRV were the agents most often detected in co-infections during the first research period (2019–2020). During the second research period (2020–2021), more co-infections were recorded with other viruses such as HMPV, HPIV, HCoV—and also HRV. During the third research period (2021–2022), the largest number of infections detected was with HRV (Figure 4C).

The seasonal coronavirus, parainfluenza virus, bocavirus, rhinovirus, and adenovirus were the most often detected in co-infections pairings for all three research periods from 2019 to 2022 (Figure 4D).

### 3.6. Analysis of the Clinical Features of ARI Infections

To assess the clinical features of ARIs, we analyzed 2659 medical record cards: 754 in 2019–2020, 933 in 2020–2021, and 972 in 2021–2022. We analyzed disease severity (based on the physician’s assessment) and such clinical signs as hyperthermia, hypoxia, and necessity for admission to an intensive care unit (ICU) (Table 1).

A severe course of disease was observed in 44% of hospitalized patients, while average course severity was registered among 56% of patients. We detected a decrease in severe cases the older the children were (aged 0–2 years [48.8%] vs. aged 3–6 years [42.4%] vs. aged 7–17 years [28.3%]) (Chi-square test, *p* < 0.05). It is interesting to note that severe courses of co-infections were registered more often compared to courses of mono-infections: 57.9% and 45.1%, respectively (χ^2^ = 12.07, *p* < 0.05). In our study the least severe courses of illness were observed in cases of influenza virus infection (26.1%; 107/410) compared to infection with other respiratory viruses.

Hospitalization in an ICU was required for only 8.1% of infected children and adolescents. The age distribution was 54.8% (125/228) among children aged 0–2 years; 29.8% (68/228) among children aged 3–6 years; and 15.4% (35/228) among children aged 7–17 years. The detection rate of common respiratory viruses in hospitalized patients admitted to an ICU was significantly higher than in those who did not need an ICU (72.8% and 64.1%, respectively; χ^2^ = 6.94, *p* < 0.05). We did not find any significant differences in the frequency of hospitalization to an ICU for patients with mono- or co-infections (10.0% and 9.0%, respectively). Patients with COVID-19 were admitted to an ICU less frequently (by 2.1%), while the other ARI incidences in children leading to admission to an ICU were 5.7% of those infected with HPIV, 6.8% with HIFV, 11.1% with HCoV, 11.4% with HRV, 9.6% with HMPV, 12.7% with HAdV, 13.0% with HBoV, and 13.5% with HRSV.

Hyperthermia (i.e., body temperature higher than 39 °C) was observed in 52.7% of child patients among whom those who suffered more often were aged 3–6 years (61.3%) compared to children aged 0–2 years (48.3%) (χ^2^ = 34.86, *p* < 0.05) and those aged 7–17 years (54.1%) (χ^2^ = 5.98, *p* < 0.05). It is remarkable that hyperthermia was observed significantly more often in cases of influenza infection (by 80.8%).

Hypoxia was observed in 20.7% of children: distinctions were not registered among the age groups. We observed that children with co-infections significantly more often suffered from hypoxia compared to those with mono-infections (32.5% vs. 23.7%, respectively) (χ^2^ = 7.79, *p* < 0.05). In addition, significantly less hypoxia was observed in children with influenza (15.0%) and COVID-19 (13.4%) compared to other infections.

## 4. Discussion

According to the healthcare authorities, the long-term average indicator of ARI incidence among the population in Russia amounted to 20,754 per 100,000 people during the period 2010–2019, subsequently rising to 22,710 per 100,000 people in 2020, and increasing further to 26,252 per 100,000 people in 2021. The largest number of ARI cases occurred among children younger than 7 years [2,7,8,9]; among children and adolescents in 2019, the prevalence rate was 71.6%, with incidences increasing by about 15% in the following years (in 2020—59,003 per 100,000 child population, and in 2021—68,063 per 100,000 child population). During the two epidemic seasons 2020–2021 and 2021–2022, an early onset of the epidemic increase in the incidence of ARIs, including SARS-CoV-2, was noted compared to the three previous epidemic seasons [10]. In our study, which was carried out on the basis of data received from two Novosibirsk Children’s Municipal Clinical Hospitals (No 3 and No 6), nasal and throat swab samples were collected from 3190 patients for testing for HIFV, HRSV, HRV, HPIV, HCoV, HMPV, HBoV, HAdV, and SARS-CoV-2 by real-time PCR in order to analyze changes in the epidemic patterns of common respiratory viruses among hospitalized children and adolescents.

We observed a dramatic variability in ARI among children over three periods: in 2019–2020, the main agents circulating were HIFV, HRSV, and HPIV; in 2020–2021, HMPV, HRV, and HCoV dominated with a much lower incidence of SARS-CoV-2, a sharp decline in HRSV, and an absence of HIFV; during the last research period (2021–2022), the most numerous agents were HRSV and SARS-CoV-2, while there was a rise in the incidence of HRV and a re-emergence of HIFV (interestingly, there was an absence of HMPV in this period that had dominated in the previous period). It is also noteworthy that during the previous pandemic of 2009, which was caused by the influenza A(H1N1)pdm09 virus, a prevalence of HMPV among children was also detected [11]. Thus, it seems that HIFV and HRSV have an integrative relationship in the etiology of ARIs [12]. In our research, similar annual fluctuations in HIFV and HRSV during the pre-pandemic and continuing pandemic periods were demonstrated. The investigation of relationships between the epidemic curves of HPIV, HBoV, and HAdV was limited by the small proportion of positive tests. The endemic nature of HRV appeared to be unaffected by the circulation of other respiratory viruses, which supports previous observations [5,12]. The seasonal occurrence of agents may be dependent upon many factors; further studies are required for a greater understanding of the interaction between respiratory viruses that will help to predict respiratory virus epidemics.

Previous studies have shown that the SARS-CoV-2 pandemic affected the circulation of other seasonal viruses, the infection of children of different ages, and the clinical manifestations of ARI [5,6,13,14].

In April 2020, shortly after the announcement of the COVID-19 pandemic by the WHO [15], there was a sharp decrease in the number of laboratory-confirmed cases of HIFV [5,13,16]. It was suggested that the restrictive measures aimed at stopping the transmission of SARS-CoV-2 significantly contributed to the reduction of morbidity and the end of the epidemic flu season [17].

Several studies have demonstrated respiratory virus interactions during circulation with SARS-CoV-2 [14,17,18]. In our studies, variants of viral co-infections were also shown, among which the most numerous were HRSV, HPIV, HBoV, HCoV and HAdV in the 2019–2020 period; HMPV, HPIV, HCoV, and HRV in the 2020–2021 period; and HRV, HPIV, HRSV, HAdV, and HCoV in the 2021–2022 period. The lowest levels of co-infections were detected for SARS-CoV-2 and HIFV during all seasons investigated. Of interest was the detection of an increase in dual and triple co-detections in the form of multiple identical viral combinations during the second wave of the pandemic. It was also found that cases of SARS-CoV-2 co-infection are possible with HRV, HRSV, HAdV, HMPV, and HPIV.

In our study, we noted an increase in the incidence of COVID-19 only during the third research period (2021–2022), the peak incidence of which occurred from January to March 2022. The intensive development of the COVID-19 epidemic process on a global scale has created favorable evolutionary conditions for the emergence of new genetic variants of the virus. The first significant mutation, the variant B.1.1.7 (Alpha), was detected in the UK in December 2020. In April 2021, the variant B.1.617.1/B. 1.617.2 (Delta/Kappa) was detected, and in November 2021, a new variant B.1.1.529 (Omicron) emerged. It is probable that the increase in the registration of SARS-CoV-2 cases among hospitalized children was due to infection with Omicron. Recent studies revealed that children and adolescents show the most pronounced loss of cross-neutralization against Omicron [19,20].

We observed that co-infections did not affect the frequency of hospitalization in an ICU. However, patients suffered from hypoxia more often when they were co-infected. In our research, no significant differences were observed among children infected with SARS-CoV-2 belonging to different age groups, which was also shown in the study of Hansen and coauthors [21] We did not find any sex differential responses and outcomes of infectious diseases in the current research, while some authors highlight the contribution of gender-associated factors to affect the outcomes of many respiratory viral infections [22]. These conclusions were based on the analysis of 2659 pediatric medical records.

This cohort study has revealed that during the pre-pandemic and pandemic periods, there were dramatic fluctuations in the common respiratory viruses registered among hospitalized patients 0–17 years old. The most dominant virus in each research period differed: HIFV in 2019–2020, HMPV in 2020–2021, and HRSV in 2021–2022. It is interesting to note that certain viruses were not detected during a particular research period (i.e., HIFV in 2020–2021 and HMPV in 2021–2022) but re-emerged one year later (HIFV and HRSV in 2021–2022). Viral co-infection was significantly more frequently detected during the pandemic period of 2020–2021. In our research, virus–virus interactions were registered between SARS-CoV-2 and HRV, HRSV, HAdV, HMPV, and HPIV.

## Figures and Tables

**Table 1:** Data of disease severity among patients aged 0–17 years hospitalized during 2019–2022.

Virus	Hyperthermia (%)	Hypoxia (%)	Severe Disease (%)	ICU ^1^ (%)
total	1486 (52.7)	583 (20.7)	1234 (44.0)	228 (8.1)
HIFV	333 (80.8)	62 (15.0)	107 (26.1)	28 (6.8)
HRSV	123 (40.6)	97 (32.0)	175 (57.9)	41 (13.5)
HRV	112 (35.7)	89 (28.3)	163 (52.1)	37 (11.4)
HPIV	115 (50.2)	50 (21.8)	119 (51.7)	13 (5.7)
HCoV	73 (42.4)	40 (23.2)	72 (42.1)	35 (11.1)
HMPV	144 (44.4)	126 (38.9)	181 (56.0)	16 (9.6)
HBoV	31 (44.9)	26 (37.7)	38 (57.6)	9 (13.0)
HAdV	65 (67.0)	22 (22.7)	60 (61.8)	8 (12.7)
SARS-CoV-2	76 (53.5)	19 (13.4)	70 (50.7)	3 (2.1)
Co-infections	93 (44.5)	68 (32.5)	121 (57.9)	21 (10.0)
Mono-infections	875 (54.1)	383 (23.7)	726 (45.1)	145 (9.0)

Note: ^1^—ICU—intensive care unit. Abbreviations: HIFV—influenza A and B viruses; HRSV—respiratory syncytial virus; HRV—rhinovirus; HPIV—parainfluenza virus types 1–4; HCoV—alphacoronaviruses (NL63/229E) and betacoronaviruses (OC43/HKU1); HMPV—metapneumovirus; HBoV—bocavirus; HAdV—adenovirus; and SARS-CoV-2—severe acute respiratory syndrome coronavirus 2. All information is presented as numerical and percentages data of medical records analyzed of patients who were positive with acute respiratory infections during 2019–2022.

## Data Availability

The data is unavailable due to privacy.

## Supplementary Materials

The following supporting information can be downloaded at: https://www.mdpi.com/article/10.3390/v15040934/s1, Table S1: Comparison of two virus groups.
