# Supplementary material for: Changes in the Etiology of Acute Respiratory Infections among Children in Novosibirsk, Russia, between 2019 and 2022: The Impact of the SARS-CoV-2 Virus
Source: Viruses. 2023 Apr 9;15(4):934. doi: 10.3390/v15040934 (PMC10141072; doi:10.3390/v15040934)
Supplement: Supplementary file 1 [file viruses-15-00934-s001.zip › viruses-2273371-supplementary.pdf]

**Table S1.** Comparison of two virus groups.

| 2019–2020 | Comparison of two virus groups | | 2020–2021 | Comparison of two virus groups | | 2021–2022 | Comparison of two virus groups | |
|---|---|---|---|---|---|---|---|---|
| | $\chi^2$ | *P*-value | | $\chi^2$ | *P*-value | | $\chi^2$ | *P*-value |
| HIFV vs HRSV | 16.52 | <0.01 | HMPV vs HRV | 46.38 | <0.01 | HRSV vs SARS-CoV-2 | 12.87 | <0.01 |
| HIFV vs HRV | 162.21 | <0.01 | HMPV vs HCoV | 76.8 | <0.01 | HRSV vs HIFV | 32.38 | <0.01 |
| HIFV vs HPIV | 134.57 | <0.01 | HMPV vs HPIV | 121.81 | <0.01 | HRSV vs HRV | 36.53 | <0.01 |
| HIFV vs HCoV | 270.7 | <0.01 | HMPV vs HBoV | 243.23 | <0.01 | HRSV vs HAdV | 110.88 | <0.01 |
| HIFV vs HMPV | 311.63 | <0.01 | HMPV vs HAdV | 342.37 | <0.01 | HRSV vs HPIV | 112.73 | <0.01 |
| HIFV vs HBoV | 203.85 | <0.01 | HMPV vs SARS-CoV-2 | 356.2 | <0.01 | HRSV vs HCoV | 167.56 | <0.01 |
| HIFV vs HAdV | 243.57 | <0.01 | HMPV vs HRSV | 356.2 | <0.01 | HRSV vs HBoV | 167.56 | <0.01 |

Abbreviations: HIFV - influenza A and B viruses; HRSV - respiratory syncytial virus; HRV - rhinovirus; HPIV - parainfluenza virus types 1–4; HCoV - alphacoronaviruses (NL63/229E) and betacoronaviruses (OC43/HKU1); HMPV - metapneumovirus; HBoV – bocavirus; HAdV – adenovirus; and SARS-CoV-2 - severe acute respiratory syndrome coronavirus 2.
